# Validating a case definition for adult asthma in primary care electronic medical records

**DOI:** 10.1038/s41533-020-0181-3

**Published:** 2020-06-05

**Authors:** Andrew J. Cave, Boglarka Soos, Christina Gillies, Neil Drummond, Anh N. Q. Pham, Tyler Williamson

**Affiliations:** 1 Department of Family Medicine, University of Alberta, Edmonton, AB Canada; 2 Department of Family Medicine, University of Calgary, Calgary, AB Canada; 3 Department of Community Health Sciences, University of Calgary, Calgary, AB Canada; 4 Department of Agricultural, Food & Nutritional Science, University of Alberta, Edmonton, AB Canada; 5 School of Public Health, University of Alberta, Edmonton, AB Canada

**Keywords:** Asthma, Epidemiology

## Abstract

Although asthma is one of the most common chronic conditions affecting Canadians, its epidemiologic characteristics and burden in primary care contexts are poorly understood. The aim of this study was to develop and validate a case definition to identify adults with asthma who consult family physicians and to estimate the prevalence of asthma in that setting in Canada. This validation study utilized a database of electronic medical records (EMRs) from the Southern Alberta Primary Care Research Network, a node of the Canadian Primary Care Sentinel Surveillance Network (SAPCReN-CPCSSN). The population included patients over age 17y of any gender and health status who had visited an SAPCReN-CPCSSN primary care provider during the period December 1, 2014−December 31, 2016. The validation of the case definition involved comparing a case-finding algorithm to caseness determined by an expert physician review of the records of 1000 patient in the CPCSSN database. The case definition, which included the ICD-9 code 493 and asthma-related text words, had 83.33% sensitivity (95% CI: 63.61–93.88%), 99.28% specificity (95% CI: 98.51–99.67%), a positive predictive value of 74.07% (95% CI: 55.03–87.14%), and a negative predictive value of 99.59% (95% CI: 98.93–99.86%). The prevalence of adult asthma in CPCSSN primary care practices in southern Alberta was 4.20% (95% CI: 4.09–4.31). The strong validation metrics suggest that this case definition is valid for both clinical and research purposes. The validated case definition may be used to improve patient care and improve understanding of the prevalence and burden of asthma in primary care in Canada.

## Introduction

Asthma is one of the most common chronic conditions affecting Canadians, with over 8% of the population aged 12 and over reporting physician-diagnosed asthma^1^. For most adult patients, asthma is diagnosed and managed in primary care^2^. In this context, electronic medical records (EMRs) are an accessible and comprehensive source of data about the condition. Studies utilizing EMR data to determine prevalence and epidemiologic characteristics of asthma in adults are few in number and frequently differ in terms of methodology, sample characteristics, and case definition criteria^3^. Improving the rigor of the case definition for adult asthma in the Canadian primary care context through the use of a clinical database is therefore very important.

The Canadian Primary Care Sentinel Surveillance Network (CPCSSN) has developed a process that enables data from EMR databases to be extracted, cleaned, and merged into a single national primary-care data set^4^. The use of these data is intended to enhance patient care by improving understandings of the epidemiology of chronic illnesses^4^. A case definition for pediatric asthma has previously been validated in the CPCSSN database^5^; however, as asthma presents differently for adults, it cannot be assumed that it is also valid for adults. The purpose of this study was to develop and validate a case definition and case-finding algorithm to identify adults with asthma who consult family physicians and to provide an estimate of asthma prevalence among community-dwelling primary care patients.

## Results

### Inter-rater agreement

Inter-rater reliability was computed by assessing the strength of agreement among 100 random SAPCReN-CPCSSN records assessed by all four reviewers. The Fleiss Kappa coefficient was 0.71 (95% CI: 0.64–0.78, two-sided *p* < 0.001), indicating “substantial agreement” between the four reviewers^6^.

### Case definition and validation metrics

The case-finding algorithm was adjusted several times in an iterative process and the case definition that best approximated the reference standard was selected. The final case definition is presented in Table 2. A patient was determined to have asthma if two occurrences of the text “asth*” or the International Statistical Classification of Diseases and Related Health Problems, Ninth Revision, (ICD-9) code 493 were found in the EMR Billing or Encounter Diagnosis tables during the final 24 months of the study period. A single instance of either indication in the EMR Health Condition table was sufficient to establish that the patient had asthma. Text matches that convey an uncertain diagnosis of asthma, indicated by the word “query” or a question mark (?), were excluded. This case definition had 83.33% sensitivity (95% CI: 63.61–93.88%), 99.28% specificity (95% CI: 98.51–99.67%), a positive predicted value (PPV) of 74.07% (95% CI: 55.03–87.14%), and a negative predicted value (NPV) of 99.59% (95% CI: 98.93–99.86%).

### Prevalence

When the case-finding algorithm was applied to the SAPCReN-CPCSSN database, the prevalence estimates for adult asthma in southern Alberta were 4.20% (95% CI: 4.09–4.31). Specifically, 5014 asthma cases among the 119,416 individuals over the age of 17y had contact with a CPCSSN sentinel between January 1, 2015 and December 31, 2016. The difference in the age and sex distribution of patients randomly selected for the study sample and the general population in Canada, based on the 2016 census, is not statistically significant (*χ*^2^ = 40.7, 35 degrees of freedom, two-sided *p* = 0.23). This suggests that our results may be generalizable to the Canadian population.

## Discussion

The case-finding algorithm demonstrated excellent specificity and NPV, as well as good sensitivity and PPV for identifying adults with and without asthma in the SAPCReN-CPCSSN database. Given the high sensitivity and specificity values, the case-finding algorithm may be useful for epidemiological purposes and may also be used in population surveillance to identify asthma prevalence and distributions in the community. The excellent NPV, along with the relatively high PPV (especially in the context of a fairly low calculated prevalence estimate), indicate that the case-finding algorithm is appropriate for use in clinical practice to identify individual asthma patients to improve their care. The algorithm may also be useful for the development of cohort studies as well as for the identification of patients for clinical trials.

It is important to note that the prevalence estimate for adult asthma obtained from our study is lower than the estimated prevalence used in the sample size calculation. As a result, the 95% confidence intervals for the sensitivity and PPV are wider than anticipated. However, our prevalence estimate aligns well with that of Tonelli et al.^7^, which suggests our results are underpowered but may be generalizable beyond the study sample. A study with a larger sample size than 1000 is recommended.

Case-finding algorithms have previously been validated for a number of chronic conditions in the CPCSSN database, including chronic obstructive pulmonary disease (COPD)^5,8^. These validation studies utilized a similar process whereby original patient charts were audited by primary care physicians to determine whether patients had any of the CPCSSN indexed conditions and then compared to a CPCSSN case definition diagnosis. This is the second study to use CPCSSN records—rather than original EMR charts—to validate a disease diagnosis. A previous validation study of a pediatric asthma case definition yielded similar metrics to the current study in terms of sensitivity (87.4%), specificity (98.6%), and NPV (97.9%), but had a higher PPV (91.2%). The current study further demonstrates the potential and feasibility of using CPCSSN algorithms to identify patients in primary-care practices for clinical and research purposes.

In Canada, the majority of studies seeking to validate a diagnosis of adult asthma have used health administrative data, patient survey, and professional diagnosis of asthma by a health-care professional. In Ontario, a validated case definition for asthma using health administrative data required an asthma diagnoses in two or more ambulatory care visits and/or one or more hospitalizations for asthma in a 2-year period and yielded sensitivity of 83.8%, specificity of 76.5%, PPV of 61.5%, and NPV of 91.3%^9^. This study considered individuals aged 19−80 and oversampled those with respiratory conditions to ensure the algorithm was able to distinguish between asthma, COPD, and asthma-related conditions. In Quebec, asthma diagnosis derived from a physician billing database was compared to the documented diagnosis in a patient’s medical chart by family physicians^10^. Adopting a definition of one or more diagnoses of asthma over a 12-month period, the study yielded a PPV of 67.0% and NPV of 99.0% for adults aged 16–44, and a PPV of 60.0% and NPV of 100% for adults aged 45–80. In Manitoba, one study compared physician payment claims in a provincial health administrative database with survey data regarding the prevalence of asthma in individuals aged 20–44 and found only moderate (*k* = 0.45−0.50) agreement^11^. Estimates of the prevalence of asthma ranged from 4.0 to 8.6% and was dependent on the metric captured by the survey and the time period used when exploring the claims database. Finally, Tonelli et al. applied Gershon’s validated algorithm^9^ to a identify patients with chronic conditions and multimorbidity using inpatient and outpatient claims and utilization data. Their work indicated the estimated prevalence of adult asthma was 2.3% in a population of adults residing in Edmonton, Alberta, Canada between April 2008 and March 2009.

Our study expands upon previous studies by validating the case definition using individualized electronic patient health information from the primary care setting and including elements such as encounter diagnoses and the patient’s problem list. SAPCReN-CPCSSN data provide more granular details about a patient’s health status and may yield results more generalizable and accurate than studies utilizing primary care administrative data or survey data.

A strength of the present study was the utilization of SAPCReN-CPCSSN records, which allowed the study access to a large sample of adult patients within the southern Alberta primary care population. This approach also provided access to consistent data, anonymity for all patients, and resulted in both time and cost-efficient access to data. However, the expert reviewers did not have access to unstructured clinical notes or referral letters for reasons associated with confidentiality and patient anonymity. In addition, the quality of the data collected was dependent on the data recorded in the family physicians’ offices. Our study yielded a relatively low PPV (74.07%) because of instances in which the case-finding algorithm identified patients as having asthma but the reviewers did not. We hypothesize that asthma may have been underdiagnosed by the reviewers as a result of the quality and types of information available to reviewers in CPCSSN records. Finally, it may be argued that our study may have limited applicability as the case-finding algorithm is limited to CPCCSN data and may not be implemented on “raw” EMR data which has not undergone CPCSSN processing. However, as we have shown that our results are generalizable to the Canadian population, the algorithm should be applicable in the presence of adequate clinical records.

This study provides a valid case definition and case-finding algorithm for the identification of adults with asthma in the primary care setting in Canada. The validated case definition may be used to enhance our understanding of the burden of asthma in the adult population through improved surveillance, quality improvement and research. It may also lead to improved asthma care by facilitating better patient identification, monitoring, and management. A continued focus on the development and use of validated case definitions and case-finding algorithms in EMR databases is an important step in ensuring the accurate measurement and understanding of the prevalence and burden of chronic noncommunicable diseases in Canada.

## Methods

### Data source and study population

The CPCSSN is a network of 12 practice-based primary care research networks across Canada. CPCSSN has established national as well as network-specific databases of primary care patient health data for use in surveillance, research, and quality improvement studies^12^. The CPCSSN databases contain patient records routinely extracted from the EMRs of participating sentinel providers (e.g., family physicians, nurse practitioners, and pediatricians), which have been processed through computerized coding and cleaning algorithms^4^. The following information is extracted about patients and included in the CPCSSN database: demographics, encounter dates and types, health conditions, physical examinations, risk factors, procedures, prescribed medications, referrals, and laboratory investigations. During the data cleaning process, invalid entries are deleted and the data are standardized using text matching algorithms which map prescribed medications to Anatomical Therapeutic Chemical (ATC) Classification codes, laboratory variable names to Logical Observation Identifiers Names and Codes (LOINC) codes, and medical diagnoses to ICD-9 codes. A de-identification process is applied to free text to render the CPCSSN data anonymized. Clinical notes, PDF documents, and directly identifiable patient information, including names and contact information, are not extracted (or “redacted”) from the patient’s EMR. CPCSSN securely collects and combines data shared from the primary care practice-based research networks and stores the data in a secure, central data repository at Queen’s University (Ontario, Canada)^4^. We obtained a waiver of individual patient consent because our sample consisted of CPCSSN records identified by CPCSSN identification number only. Approval for the study was received from the Health Research Ethics Board at the University of Alberta (Pro00072496) and the Conjoint Health Research Ethics Board (CHREB) at the University of Calgary (REB17-1710).

**Fig. 1:**
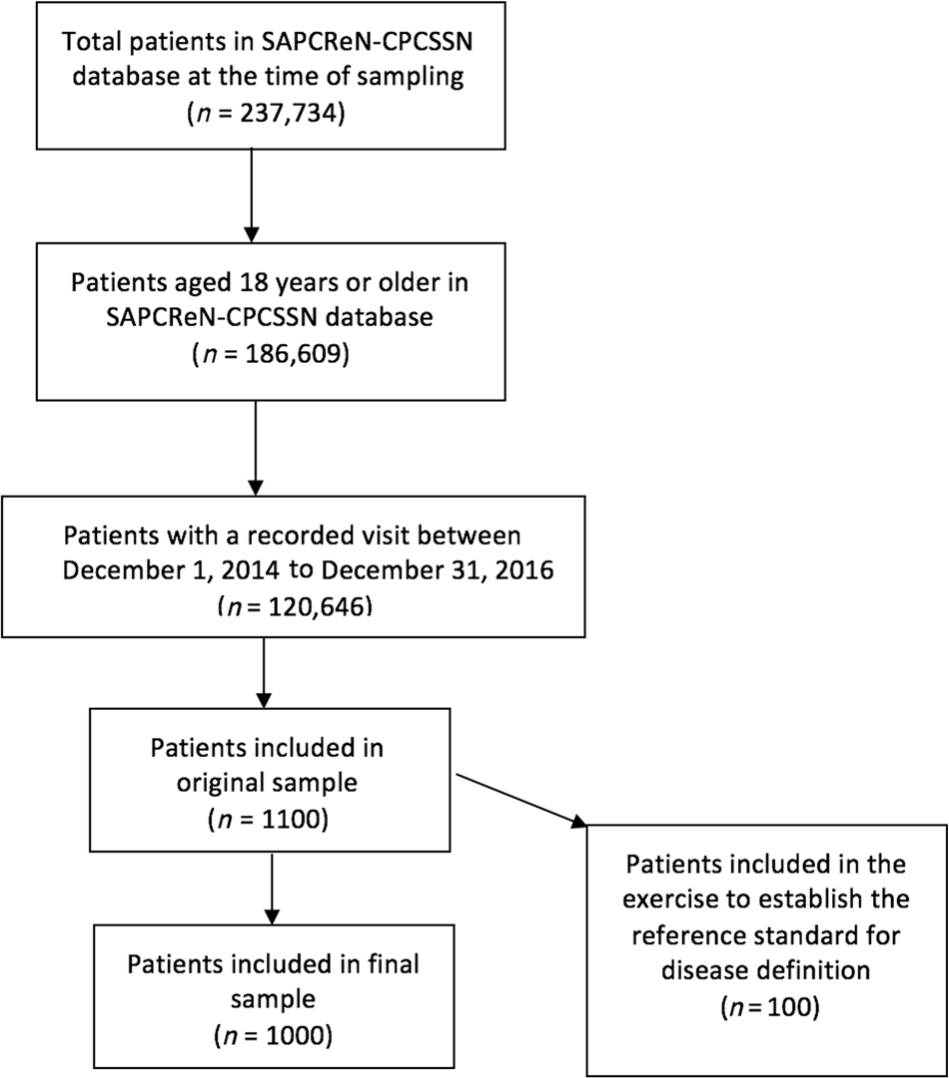
Study flow chart. Total number of patients in the SAPCReN network, those included as adults, and those considered active patients with a visitin the three year timeframe.

The present study utilized data from the Southern Alberta Primary Care Research Network (SAPCReN), the node of CPCSSN hosted by the Department of Family Medicine at the University of Calgary. SAPCReN-CPCSSN extracts data from primary care providers, nurse practitioners, and community pediatricians in Southern Alberta. Participation in SAPCReN-CPCSSN is voluntary. At the time of this study, the SAPCReN-CPCSSN database included 220 sentinel providers and the records of more than 237,000 patients. The study sample consisted of random, de-identified SAPCReN-CPCSSN records of 1000 active adult patients (over age 17y) of any gender and health status (Fig. 1). An “active” patient was defined as an individual who received care from a family physician participating in SAPCReN-CPCSSN between December 1, 2014 and December 31, 2016. A comparison of the basic demographics of the study sample and the sampling frame is presented in Table 1. Data were retrieved dating from January 1, 2014 to December 31, 2016. This sample size was determined on the assumption of a disease prevalence of 10% and sensitivity >70% with a 95% confidence interval that has a width of no more than 20%. Sensitivity values of 70% were considered as the minimum threshold for a valid case definition.

**Table 1:** Demographic statistics for the study sample and the sampling frame.

Demographic	Sample (%)	Sampling frame (%)
Sex		
Male	47.5	45.2
Female	52.5	54.8
Age		
18–29	16.4	17.1
30–39	15.9	17.9
40–49	17.5	16.6
50–59	17.8	18.8
60–69	16.7	15.8
70–79	8.8	8.6
80+	6.9	5.3

### Establishing the reference standard

To establish the reference standard for disease definition, two respirologists and two family physicians from the Family Physicians Airways Group of Canada independently reviewed the full SAPCReN-CPCSSN records for a sample of 100 patients and assessed “caseness” (that is, asthma or not asthma) in each, based on current literature and clinical experience. As a cohesive group, the reviewers developed a list of criteria to consider, including patient age, gender, diagnostic labels (e.g., “asthma” text and/or ICD code 493) in the billing entry, encounter text and problem list; the use of medications (e.g., inhaled corticosteroids); and diagnostic tests (e.g., spirometry). Fleiss’ Kappa was employed to measure the strength of agreement in the initial review of 100 random SAPCReN-CPCSSN records among the four reviewers.

Each reviewer subsequently independently assessed a new random sample of 250 records to create the total sample of 1000. To find evidence of active asthma in the CPCSSN record, the reviewers had access to all data dated January 1, 2014−December 31, 2016. In cases where there was uncertainty, the SAPCReN-CPCSSN records were sent to the other three reviewers to assess “caseness” and the majority answer was selected as the decision. The method of using CPCSSN-processed data to identify the set of reference cases has been proven to be an acceptable substitute for raw EMR data^13^.

### Case definition development and case-finding algorithm development

An operational case definition for adult asthma was developed by the study team based on the available and applicable data fields found in the SAPCReN-CPCSSN database. The case definition used a combination of ICD-9 codes and textual variables drawn from various sections of the EMR, including billing, encounter diagnosis, health conditions, and prescribed medications. We then developed a computerized case-finding algorithm using the SAPCReN-CPCSSN 2016-Q4 data for the same sample of 1000 patients reviewed by the expert physicians. ICD-9 codes, textual words, and medications relevant to asthma were considered as potential components of the case-finding algorithm, and the number of instances of each indicator over the study period was taken into account. The case-finding algorithm was adjusted several times in an iterative process, until its output generated sets of cases and non-cases which appeared to most closely approximate the sets of reviewer-defined cases and non-cases.

### Statistical analysis

The case definition was validated by comparing the algorithm results against the physician record review. Validation of the case definition involved the creation of a two-by-two table (Table 2) and the calculation of sensitivity, specificity, PPV, and NPV.

**Table 2:** Final case definition and case-finding algorithm.

Billing^a^	Encounter diagnosis^b^	Health condition^c^
At least two occurrences of the following ICD-9 code:	At least two occurrences of the following ICD-9 code:	At least one occurrence of the following ICD-9 code:
493—asthma	493—asthma	493—asthma
	OR	OR
	At least two occurrences of the following text:	At least one occurrence of the following text:
	- asth*	- asth*
	The following text are excluded:	The following text are excluded:
	- asthma*query - query*asthma - asthma*? - ?*asthma	- asthma*query - query*asthma - asthma*? - ?*asthma

^a^The Billing table contains all billing data submitted to the province.

^b^The Encounter Diagnosis table contains the diagnoses recorded during an encounter with the patient.

^c^The Health Condition table (also known as the Problem List) records important details from a patient’s medical history, including diagnoses that require active monitoring or impact decisions related to patient care.

To estimate the 2-year period prevalence of adult asthma, the case definition was applied to the sampling frame. All individuals over the age of 17y with at least one encounter with a SAPCReN-CPCSSN sentinel between January 1, 2015 and December 31, 2016 were included. Prevalence estimates were calculated as rate per hundred (cases/total sample × 100).

A chi-square test was used to determine if the age and sex distribution of the study sample was different from the Canadian population, using data from the 2016 census^14^.

Python 2.7.10 was used for data processing, implementation of the algorithm, and analysis, and SQLite was used for database management. STATA SE 15 was used to calculate 95% confidence intervals for the estimated proportions for the analysis.

### Reporting summary

Further information on research design is available in the Nature Research Reporting Summary linked to this article.

## Supplementary information

Reporting Summary

## Data Availability

The data that support the findings of this study are available from CPCSSN but restrictions apply to the availability of these data, which were used under license for the current study, and so are not publicly available. Data are, however, available from the corresponding author (A.J.C.) upon reasonable request and with permission of CPCSSN.

## Code availability

The Python programming code used for this study is available from the corresponding author (A.J.C.) upon reasonable request.
